# Management of an Endodontic-Periodontal Lesion in a Maxillary Lateral Incisor with Palatal Radicular Groove: A Case Report

**DOI:** 10.7508/iej.2016.02.014

**Published:** 2016-03-20

**Authors:** Aidin Sooratgar, Mehdi Tabrizizade, Maryam Nourelahi, Yasin Asadi, Hosein Sooratgar

**Affiliations:** a *Department of Endodontics, Dental School, Tehran University of Medical Sciences and Health Services, Tehran, Iran; *; b *Department of Endodontics, Dental School, Shahid Sadoughi University of Medical Sciences and Health Services, Yazd, Iran;*; c *Department of Periodontics, Dental School, Semnan University of Medical Sciences and Health Services, Semnan, Iran;*; d *Department of Periodontics, Dental School, AJA University of Medical Sciences and Health services, Tehran, Iran;*; e *Department of Restorative Dentistry, Dental School, Gilan University of Medical Sciences and Health Services, Tehran, Iran*

**Keywords:** Endodontic-Periodontal Lesion, Maxillary Lateral Incisor, Palatoradicular Groove, Periodontal Regeneration

## Abstract

The radicular groove is a developmental anomaly that predisposes the tooth to periodontal break-down. Sometimes the situation becomes more complicated by pulp necrosis and development of a combined endodontic-periodontal lesion which is a diagnostic and treatment challenge for the clinician. This report presents the successful management of an endodontic-periodontal lesion in a maxillary lateral incisor that has a developmental palatal radicular groove using a combination of nonsurgical endodontic therapy and periodontal regenerative techniques.

**Conclusion:**

The combination of nonsurgical endodontic and periodontal regenerative treatment is a predictable method in treating combined endodontic-periodontal lesions caused by palato-gingival groove.

## Introduction

The palatal radicular (palatoradicular) groove is a linear depression or groove that begins at the central fossa of the maxillary incisors, extends over the cingulum and continues specifically to the root surface [1] and possibly reaches the apex [2]. Palatal radicular grooves are different in depth and complexity. They are classified into three types based on their severity: *Type I*; the groove is short but does not extend beyond the coronal third of the root, *type II*; the groove is long reaching beyond the coronal third of the root but shallow, corresponding to a normal or simple root canal and *type III*; the groove is long, deep and extends beyond the coronal third of the root, corresponding to a complex root canal system [3].

Lee *et al. *[1] believe that the radicular groove represents a minimal in-folding of the enamel organ and Hertwig's epithelial root sheath cells during odontogenesis and therefore it is analogous to the pathogenesis of dens invaginatus. The prevalence of palatal groove has been reported to be 2.8-8.5% [4]. Radicular groove is a locus for plaque accumulation and it provides a potential pathway for microorganisms to penetrate into deeper parts of the periodontium and cause local inflammation [5]. Whenever the periodontal tissue attachment is disrupted, a self-sustaining progressive localized periodontal pocket can develop along the groove. This pocket can reach to root apex and affect the pulp vitality and establish a combined periodontal-endodontic lesion.

Treatment of the radicular groove, include curettage of the affected periodontal tissues [6], saucerisation of the groove [7], sealing the groove with a biocompatible material [7], root canal treatment when a primary or secondary endodontic lesion is present [8] and surgical procedures (*i.e.*, guided tissue regeneration therapy, intentional replantation [4, 9, 10].

Considering the great clinical importance of the palatal radicular groove and its rare occurrence, this paper reports a case involving a primary periodontal lesion with secondary endodontic involvement in a maxillary lateral incisor with palatal radicular groove. A multidisciplinary treatment approach included both endodontic treatment and guided tissue regeneration. 

**Figure1:**
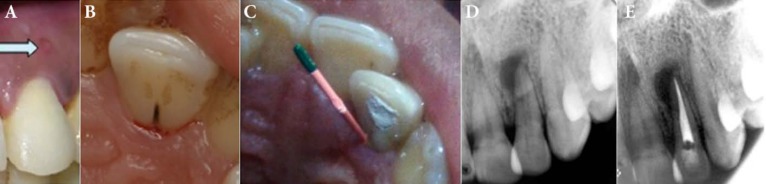
*A)* Pretreatment clinical image of the maxillary left lateral incisor with sinus tract; *B)* Palatal groove; *C)* Tracing of the radicular groove; *D)* Periradicular lucency in pretreatment radiography; *E)* Post-endodontic radiography

## Case Report

A 27-year-old female patient with good general health presented to endodontic department of Dental School, Shahid Sadooghi University of Medical Sciences, Yazd, Iran, with a chief complaint of pus discharge from buccal mucosa adjacent to maxillary left lateral incisor for the past 6 months (Figure 1A and B). The periodontal pocket was traced with #35 a gutta-percha point and revealed the extension of lesion to the apical third of the root (Figure 1C).

The patient did not mention any previous trauma to the maxillary anterior region and had no history of pain in the area. Tooth #10 had an intact crown without caries or fracture. The response to refrigerant spray and Electric pulp tester (Analytic Technology, Redmond, WA, USA) was negative while adjacent and opposite teeth responded normally. The mobility of the tooth and its response to percussion and palpation tests were within the normal limits. Periodontal examination revealed a 12-mm pocket in mid palatal region (Figure 1C). Purulent exudate was excreted from palatal sulcus which was associated with a palatal groove on cingulum of the tooth, extending to gingival sulcus. Probing depth of the buccal gingival sulcus was within normal limits. Maxillary right lateral incisor was also examined for similar condition, a shallow groove was evident but probing depth of buccal and lingual sulcus was within normal limits. Radiography showed a periradicular radiolucency involving the apical one-third of the root and a para pulpal radiolucent line that is a typical radiographic representation of the palatal groove (Figure 1D).

Based on history, clinical and radiographic examination, the lesion was provisionally diagnosed as primary periodontal lesion with secondary endodontic involvement according to Simon’s classification [11] in a maxillary lateral incisor because of a radicular groove. The patient was informed about the questionable long-term prognosis of the tooth #10 related to the length and depth of the radicular groove. Treatment options were offered to the patient including collaborative management using a combination of endodontic therapy, sealing the groove and periodontal regenerative procedure. Patient opted for this procedure and informed consent was gained. 

Endodontic treatment was started under rubber dam isolation. Cleaning and shaping of the canal was carried out using the crown-down technique up to #40 master apical ﬁle using hand K-files (Mani, Tochigi, Japan). Root canal was irrigated with 1% sodium hypochlorite. The tooth was obturated with gutta-percha (Gapadent Co Ltd, Tianjin, China) and AH-26 sealer (Dentsply, De Trey, Konstanz, Germany) using lateral condensation technique (Figure 1E). One week later the crown was restored permanently with composite resin (Z250, 3M ESPE, St Paul, MN, USA).

At one-week endodontic follow-up evaluation, the buccal sinus tract and pus drainage from palatal sulcus was eliminated and the sulcus adjacent to the palatal groove could be still probed with gutta-percha cone to a depth of 12 mm. Three-month recall showed no abnormal symptoms while palatal probing depth did not change.

**Figure 2:**
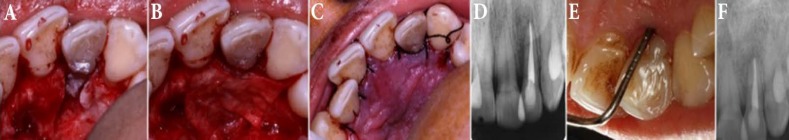
*A)* Chemical conditioning of the groove with 10% poly acrylic acid and glass ionomer; *B)* Placement of bioabsorbable collagenous membrane; *C)* Flap replacement and suturing; *D)* Six-month radiography showing partial disappearance of the radiolucency; *E)* Three-mm palatal probing depth; *F)* Two-year follow-up radiography

Upon further consultation with the periodontist, an exploratory surgery was planned. After flap reflection, a narrow palatal bony defect was noted along the root adjacent to radicular groove. Transillumination revealed no apparent cracks or fractures. The diseased granulation tissue filling the bony defect was curetted to leave the soft tissue more conducive to regeneration and the root surfaces exposed to the defect were planned with an ultrasonic scaler (Odontoson, Odonto-Wave, Fort Collins, CO, USA) and hand curettes. Also 3 mm of the root apex was resected in order to eliminate probable extra radicular microbial biofilm. Odontoplasty was performed on the lingual aspect of the root, but the groove was too deep to be totally eliminated. Chemical conditioning of the groove was performed by 10% poly acrylic acid and glass ionomer cement (Fuji I, GC Corporation, Tokyo, Japan) was applied into the groove (Figure 2A). The area was kept isolated of blood and tissue ﬂuids during the setting of glass ionomer. Then, GTR was carried out by means of 0.5 mL of decalcified freeze dried bone (DFDB) allograft with particle size of 1000×75 µm (Cenobone, Tissue Regeneration Corporation, Kish Island, Iran) and a 10×10 mm thin bioabsorbable collagenous membrane (CenoDerm, Tissue Regeneration Corporation, Kish Island, Iran) (Figure 2B). The ﬂap was approximated and continuous sling suturing was done.

**Figure 3:**
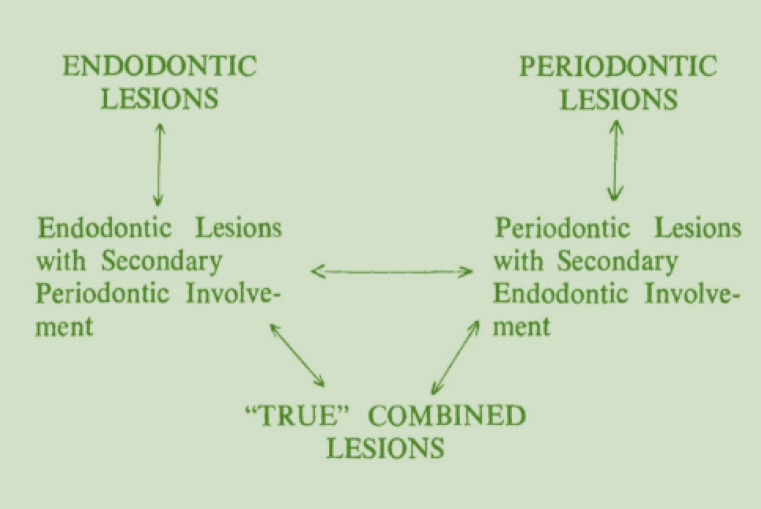
Simon’s classification

with nonresorbable 3-0 silk suture material (Figure 2C). Patient was instructed on post-surgery precautions and maintenance protocol which included rinsing with 0.12% solution of chlorhexidine twice a day.

The healing of the periodontium was uneventful. Sutures were removed 14 days after the surgery. No attempt for probing or deep scaling was made before the 6-months follow-up. A radiography taken 6 months after the surgery revealed partial disappearance of the radiolucency around the lateral incisor due to bone grafting and simultaneous bone regeneration (Figure 2D). Also palatal probing depth did not exceed 3 mm (Figure 2E). Thus a 9-mm gain of clinical attachment was achieved in an area where the original pocket depth had been 12 mm.

Follow up after 2 years showed normal periodontal and endodontic condition. Palatal probing depth was 3 mm and periapical radiographies showed bone regeneration and healing of periapical bone defect (Figure 2F). Permission was gained from the patient. 

## Discussion

Radicular grooves are more common in maxillary lateral incisors. They act as a “plaque trap” and initiating factor in localized gingivitis and periodontitis [5]. Focal attachment loss associated with radicular groove may extend apically and result in a hopeless periodontal prognosis. Long lasting deep periodontal pocket can secondarily affect pulp vitality and develop a combined endodontic-periodontal lesion [5]. The lesion was diagnosed as primary periodontal lesion with secondary endodontic involvement according to Simon’s classification (Figure 3). The teeth require endodontic treatment in addition to periodontal therapy. This situation may lead to misdiagnosis as a primary endodontic lesion. In radiographic view, the radicular groove can mimic a vertical root fracture or an extra root canal [4].

The prognosis of the affected tooth depends on the location, depth and extension of the groove, severity of the periodontal problem, accessibility of the defect and the type of groove (shallow or deep, short or long) [12].

In cases with both periodontal and endodontic involvement, like the present case, both forms of intervention are needed. Shallow grooves are located entirely on the crown and might be corrected by odontoplasty and periodontal treatment like curettage of granulation tissue. However radicular grooves associated with severe periodontal breakdown and extensive periapical lesion, need surgical intervention to eradicate inflammatory irritants and eliminating the groove [4].

Several materials such as composite resin, amalgam and mineral trioxide aggregate are utilized to fill the radicular groove [4]. MTA sets in the presence of moisture and blood, but it may be washed in trans gingival defects [13]. In this case glass ionomer cement was chosen because it has advantages like having an antibacterial effect, chemical adhesion to the tooth structure, adequate sealing ability [14, 15] and promoting epithelial and connective tissue attachment [16]. Clinical and histological studies have revealed that epithelial and connective tissue can adhere to the glass ionomer cement during the healing process [16].

Several materials were utilized to optimize regeneration of the lost periodontal and osseous structures. DFDB allograft was chosen to fill the osseous defect because of its osteoconductivity and ability to be converted into bone [8]. Several studies show that bone fill is enhanced by the addition of a graft material to GTR procedures. Finally, a collagenous membrane was placed over the defect to provide epithelial exclusion allowing periodontal ligament, cementum and bone to regenerate [17]. With this management approach there was improvement in periodontal ligament attachment and periradicular healing as seen on the follow-up radiography.

## Conclusion

The key to achieving long-term success in developmental anomalies is accurate diagnosis. Clinician’s awareness of existence of such a situation may help to avoid misdiagnosis and improper treatment of the tooth.
